# Aging specifically impairs switching to an allocentric navigational strategy

**DOI:** 10.3389/fnagi.2012.00029

**Published:** 2012-11-01

**Authors:** Mathew A. Harris, Jan M. Wiener, Thomas Wolbers

**Affiliations:** ^1^Centre for Cognitive and Neural Systems, University of EdinburghEdinburgh, UK; ^2^Centre for Cognitive Ageing and Cognitive Epidemiology, University of EdinburghEdinburgh, UK; ^3^Psychology Research Centre, Bournemouth UniversityPoole, UK; ^4^German Center for Neurodegenerative Diseases, Otto-von-Guericke UniversityMagdeburg, Germany

**Keywords:** aging, spatial navigation, strategy switching, allocentric processing, virtual reality, plus maze

## Abstract

Navigation abilities decline with age, partly due to deficits in numerous component processes. Impaired switching between these various processes (i.e., switching navigational strategies) is also likely to contribute to age-related navigational impairments. We tested young and old participants on a virtual plus maze task (VPM), expecting older participants to exhibit a specific strategy switching deficit, despite unimpaired learning of allocentric (place) and egocentric (response) strategies following reversals within each strategy. Our initial results suggested that older participants performed worse during place trial blocks but not response trial blocks, as well as in trial blocks following a strategy switch but not those following a reversal. However, we then separated trial blocks by both strategy and change type, revealing that these initial results were due to a more specific deficit in switching to the place strategy. Place reversals and switches to response, as well as response reversals, were unaffected. We argue that this specific “switch-to-place” deficit could account for apparent impairments in both navigational strategy switching and allocentric processing and contributes more generally to age-related decline in navigation.

## Introduction

Cognitive faculties deteriorate in both normal aging and dementia, and navigation abilities may be among those most severely affected. Brain areas associated with navigation, including the hippocampus and entorhinal cortex, show more extensive degradation in aging and dementia than other brain regions (Driscoll et al., 2003; Du et al., 2003, 2006), and show reduced functional activation during navigation with age (Moffat et al., 2006). Furthermore, some research has confirmed that aging does impair navigational processes specifically dependent on these areas, such as cognitive mapping (Moffat and Resnick, 2002; Iaria et al., 2009) and path integration (Allen et al., 2004; Mahmood et al., 2009; Harris and Wolbers, 2012). These navigational processes are in turn dependent on a range of more fundamental processes (memory, perception, attention, movement control, etc.) also affected by aging. Some aspects of navigation, such as retracing a learned route, seem less susceptible to aging (Jansen et al., 2010), and as many complex navigational processes decline with structural changes such as hippocampal atrophy (Nedelska et al., 2012), older people may be forced to rely on these intact simpler mechanisms (Wiener et al., submitted). However, there are still further aspects of navigation that may be affected by aging but have not yet been well explored. For example, in everyday navigation we do not usually rely solely on any single navigational process; instead we must constantly switch between various different navigational strategies, such as following a familiar route and heading towards a visible landmark. We argue that this strategy switching is fundamentally important to navigation and that a deficit may contribute heavily to navigation impairments observed in aging.

According to the noradrenaline (NA) hypothesis, strategy switching is coordinated by the prefrontal cortex (PFC) and mediated by the locus coeruleus-noradrenaline (LCNA) system in response to changes in reward contingency, monitored by the anterior cingulate cortex (ACC) and orbitofrontal cortex (OFC; Aston-Jones and Cohen, 2005). The locus coeruleus (LC) operates in two modes; high phasic-low tonic, which promotes focused single task performance, and high tonic-low phasic, which promotes behavioral flexibility, such as strategy switching (Aston-Jones and Cohen, 2005; Bouret and Sara, 2005). The LC shows extensive degradation in aging (Mouton et al., 1994; Manaye et al., 1995; Grudzien et al., 2007) and NA shows signs of depletion or dysregulation (Luine et al., 1990; Li et al., 2001; Allard et al., 2011), suggesting that we may see an impairment in strategy switching in aging. Animal research has provided some support for the NA hypothesis. For example, Tait et al. (2007) tested rats on an attentional set shifting task that involves locating a reward based on one of two strategies. Following a lesion to the dorsal noradrenergic bundle (one of the LC's two main efferent fiber bundles), rats were impaired at switching between the two strategies compared to controls. Set shifting impairments have also been observed in aged mice (Young et al., 2010; Tanaka et al., 2011), monkeys (Moore et al., 2003; Hara et al., 2012), and humans (Ashendorf and McCaffrey, 2008; Gamboz et al., 2009), demonstrating that aging and noradrenergic depletion at least have similar effects on strategy switching.

In navigation, numerous strategies are required in order to utilize a range of cues that are inconsistently available, as well as to operate on smaller and larger scales. These various strategies can be discriminated by reference frame, with some operating in relation to the body's changing orientation (egocentric), and others in relation to a static external coordinate system (allocentric). For example, using environmental cues to work out how to get to a known location relies on allocentric processing, while following a known route encoded as a series of turns depends on egocentric processing. Allocentric strategies have been demonstrated to depend on the hippocampus (O'Keefe, 1990; Hartley et al., 2003; Compton, 2004; Bohbot et al., 2007), while egocentric strategies have been associated with the caudate nucleus (Cook and Kesner, 1988; Iaria et al., 2003; Postle and D'Esposito, 2003). Hippocampal and caudal grey matter are negatively correlated, suggesting that these two areas operate in competition (Bohbot et al., 2007). It is thought that the PFC determines which of these parallel systems guides behavior (Doeller et al., 2008), supposedly as mediated by the ACC, OFC, and LCNA system, as above.

The plus maze task has been used extensively to study the use of allocentric and egocentric strategies in rats (Ragozzino, 2007; Rich and Shapiro, 2007). The task involves starting from one of two opposing arms of a plus-shaped maze and locating a reward at one of the two remaining arms. Which arm is rewarded depends on the current strategy (as well as the current start arm, which is pseudorandomised). Sometimes the subject is rewarded for finishing in a specific place, i.e., the east or west arm of the maze; at other times simply for a particular response, i.e., turning left or turning right. The task can therefore be used to study switches and reversals, much like the attentional set-shifting task, but in a navigational context. Several studies (Ragozzino et al., 1999; Rich and Shapiro, 2007; Young and Shapiro, 2009) have demonstrated impaired strategy switching, but unaffected reversals, following inactivation of regions of the medial PFC, which is both comparable to findings of studies using the attentional set shifting task, and consistent with the NA hypothesis. However, while some studies have used virtual mazes to assess spontaneous use of allocentric and egocentric strategies (Iaria et al., 2003; Bohbot et al., 2007), to our knowledge, there has not yet been any research into navigational strategy switching using human subjects.

We therefore set out to explore the effects of aging on navigational strategy switching in humans. We tested normal healthy young and old adults on a virtual version of the plus maze task. While the task is computerized, it is the same as the standard plus maze task in other respects, and still requires use of the same allocentric place-based and egocentric response-based strategies. We expected older people to exhibit a specific strategy switching impairment; performing worse than younger participants in blocks following a strategy switch, but not in those following a reversal, and not due to impaired learning of either strategy.

## Materials and methods

### Participants

Eighteen (10 female) young participants (20–29 years, mean 22.22 years) and 20 (11 female) old participants (60–84 years, mean 68.6 years) were recruited from existing databases of volunteers within the local communities of Edinburgh and Bournemouth. Most therefore had previous experience of participating in research. All participants had normal or corrected-to-normal vision and no known cognitive deficits or neurological disorders.

### Procedure

Participants provided information on their age, gender, and computer experience, before completing two computerized navigation tasks on a standard desktop computer with a 24″ widescreen monitor. In addition to age and gender information, participants were asked to rate their own experience of computers and of computer games on a nine-point scale from very inexperienced to very experienced. The first computer task, a spatial working memory task, was designed to assess memory for routes and places, as well as reward sensitivity. The second was the virtual plus maze task (VPM), designed to assess switching between different navigational strategies, such as egocentric response-based strategies and allocentric place-based strategies. Participants were made fully aware of these details of the experimental procedure, and all provided informed consent before participating. This study was approved by ethics committees at both the University of Edinburgh and Bournemouth University, and conformed to the Code of Ethics and Conduct of the British Psychological Society.

### Spatial working memory task

This task served as a control task, designed to assess the working memory processes underlying performance at the main task; primarily place recall and route recall. Place recall trials were set in a virtual environment consisting of an open field surrounded by mountain scenery, with six identical landmarks arranged in a central circle. Participants were automatically moved to three of the six landmarks, returned to the origin and reoriented, then asked to revisit the same three landmarks in any order. Route recall trials were set in a grid like maze shrouded in fog to restrict visibility. Participants were first directed along a route through five junctions by arrows appearing at each one, then asked to retrace the same route without directions. An additional aspect of this task assessed sensitivity to the reward signal used in the main task. This signal would be the only feedback participants would receive in the main task, so it was important that they were able to monitor it. Throughout place and route encoding phases, the signal (a yellow ball) would sometimes appear at a landmark or junction. While revisiting the landmarks and retracing the routes, participants also had to indicate whether or not a ball had appeared at each location. The task included 10 place recall trials and 10 route recall trials, alternating between the two types.

### Virtual plus maze task

**Figure 1:**
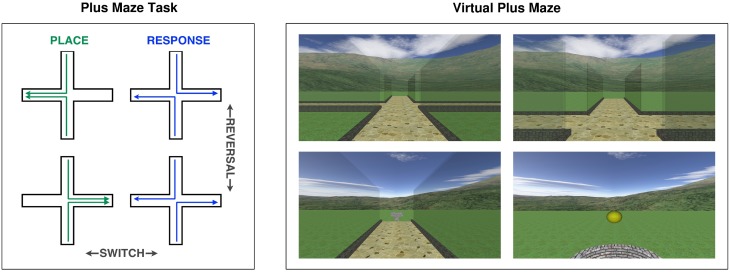
**Left:** Diagrammatic representation of the plus maze task, illustrating place and response strategies, and switch and reversal change types. **Right:** Screen images captured during the virtual plus maze task, at the start point, at the junction before turning, at the junction after turning and at the goal (showing a reward).

Our computerized adaptation of the plus maze task (Figure 1) allowed us to easily administer it to human subjects within a virtual environment. The environment consisted of a grass-textured ground plane, surrounded by continuous mountain scenery, with a central plus maze composed of curbed paths and transparent walls. The continuous mountain scenery provided visual cues from which participants could infer their orientation, without including localized landmarks that could be used as beacons. In each trial, participants started from one of two opposing start arms (in a pseudorandomised order), approached the central junction, and decided within 3 s whether to turn left or right in order to find a reward at one of the other two goal arms. A yellow ball emerged from the well at the end of the goal arm as a reward signal if the correct choice was made. Participants were rewarded either for going to the correct place (i.e., east or west, regardless of required response), or for making the correct response (i.e., left or right, regardless of heading). Between blocks of 20 trials, either a switch, a reversal or no change occurred. Switches changed the rewarded strategy from place to response, or vice versa. Reversals retained the same strategy, but changed the rewarded place or response, e.g., from east to west, or from left to right. These changes were made less predictable by the inclusion of no changes between some blocks. Participants completed a total of 320 trials, incorporating five switches and five reversals. This included three switches in one direction and two in the other, as well as three reversals for one strategy and two for the other, depending on the starting strategy, which was alternated and counterbalanced within each age group.

### Data analysis

Data analysis was performed in Matlab (Mathworks, Natick, MA). Computer experience information was combined to produce a single score. Spatial working memory task data were reduced to three scores for place recall (total correct places visited), route recall (total correct turns made), and reward sensitivity (total rewards remembered). VPM performance was assessed in terms of the number of correct trials, the number of blocks for which the strategy was learned, and the number of trials for which the learned strategy was stable. Data from blocks following no change were merged with data for the previous block. One young female participant was excluded as an outlier, as she performed more than two standard deviations below the group mean in terms of overall number of trials correct. We then used mixed model ANOVAs and *t*-tests to assess differences between age groups in numbers of trials correct, blocks learned and stable trials. *p*-values were adjusted correct for multiple comparisons according to the Bonferroni method. We also performed stepwise regression analyses to assess the contribution of age, gender, computer use, and spatial working memory task performance to measures of VPM performance.

Learning was analyzed using a Bayesian estimation procedure developed by Smith and colleagues (Smith et al., 2004, 2005, 2007), together with WinBUGs (Lunn et al., 2000) and the “matbugs” Matlab function. This approach can be used to estimate, at each time point throughout a series of trials, the likelihood of responses to all subsequent trials being correct, based on observed performance data. The point at which the lower 95% confidence interval of this estimation first exceeds and remains above the chance probability of a correct response for each individual trial (50% in this experiment) corresponds to acquisition of the reward contingency. We used this to identify whether or not the strategy was learned for each block and, if so, for how many trials the learned strategy was stable, i.e., the number of trials after the point of acquisition.

## Results

**Figure 2:**
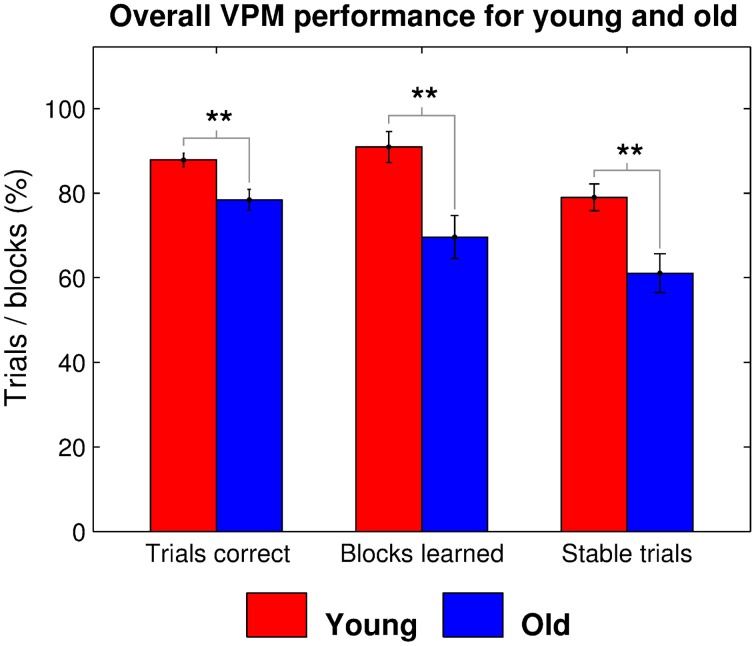
**Overall virtual plus maze task performance, as measured by the mean percentage of trials correct, blocks learned, and stable trials for young (red) and old (blue) participants**. Significant group differences are indicated at the ^*^0.05, ^**^0.01, and ^***^0.001 levels.

Older participants performed worse at the VPM in terms of overall performance, measured as the total number of trials correct (*t*_35_ = 3.052, *p* = 0.002), strategy learning, in terms of the total number of blocks learned (*t*_35_ = 3.301, *p* = 0.001), and learning speed, as indicated by the number of trials for which the learned strategy remained stable (*t*_35_ = 3.107, *p* = 0.002; Figure 2). They also reported a significantly lower level of computer experience (*t*_33_ = 3.705, *p* < 0.001) and performed worse at the spatial working memory task in terms of place recall (*t*_35_ = 4.701, *p* < 0.001) and reward sensitivity (*t*_35_ = 3.596, *p* < 0.001), although not route recall (*t*_35_ = 0.381, *p* = 0.353). We performed stepwise regression analyses to check for factors that predicted VPM performance, which confirmed that age was a significant predictor in terms of overall trials correct (β = 9.673, *p* = 0.004), blocks learned (β = 22.222, *p* = 0.002), and stable trials (β = 18.295, *p* = 0.003). However, none of the potential control variables (gender, computer use, place recall, route recall, and reward sensitivity) were retained in the models as significant predictors of any of the measures of VPM performance (Tables 1–3). Further stepwise regression analyses for each age group separately (excluding age as a predictor) maintained that none of the potential control variables were significant predictors for any measure of VPM performance. These variables were therefore not considered in further analyses.

**Table 1:** **Results of stepwise regression analysis predicting trials correct, with significant predictors (age group) highlighted in blue**.

**Trials correct**				
**Predictor**	**β**	**SE**	**In**	***p***
Age group	−9.673	3.089	Yes	0.004
Gender	3.921	3.126	No	0.219
Computer use	−0.391	0.742	No	0.602
Place recall	−0.009	0.385	No	0.981
Route recall	0.358	0.239	No	0.144
Reward sensitivity	0.510	0.301	No	0.100

**Table 2:** **Results of stepwise regression analysis predicting blocks learned, with significant predictors (age group) highlighted in blue**.

**Blocks learned**				
**Predictor**	**β**	**SE**	**In**	***p***
Age group	−22.222	6.521	Yes	0.002
Gender	7.460	6.628	No	0.269
Computer use	−1.168	1.560	No	0.460
Place recall	−0.523	0.807	No	0.521
Route recall	0.801	0.503	No	0.121
Reward sensitivity	0.897	0.644	No	0.174

**Table 3:** **Results of stepwise regression analysis predicting stable trials, with significant predictors (age group) highlighted in blue**.

**Stable trials**				
**Predictor**	**β**	**SE**	**In**	***p***
Age group	−18.295	5.738	Yes	0.003
Gender	7.032	5.814	No	0.235
Computer use	−0.558	1.381	No	0.689
Place recall	−0.241	0.714	No	0.738
Route recall	0.731	0.441	No	0.107
Reward sensitivity	0.981	0.557	No	0.088

**Figure 3:**
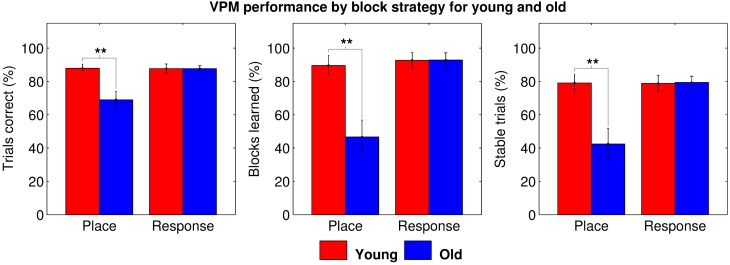
**VPM performance in terms of trials correct (left), blocks learned (middle), and stable trials (right) during place and response blocks for young (red) and old (blue) participants**. Significant group differences are indicated at the ^*^0.05, ^**^0.01, and ^***^0.001 levels following Bonferroni correction.

To explore the root of the age-related deficit in VPM performance, we split the data by block type for further analyses. We first used One-Way repeated measures ANOVAs to check for learning effects across blocks of each strategy type for each age group, but found no main effect of block order on performance (young place: *F*_(85, 4)_ = 0.790, *p* = 0.535; young response: *F*_(85, 4)_ = 0.401, *p* = 0.808; old place: *F*_(94, 4)_ = 0.828, *p* = 0.511; and old response: *F*_(94, 4)_ = 0.826, *p* = 0.512), allowing us to average performance across blocks of the same type. We then performed Two-Way mixed ANOVAs with age group as a between-groups factor and block strategy as a within-subjects factor, assigning data from place and response blocks to separate conditions. These demonstrated a significant main effect of both age (trials correct: *F*_(35, 1)_ = 9.356, *p* = 0.004; blocks learned: *F*_(35, 1)_ = 11.929, *p* = 0.001; and stable trials: *F*_(35, 1)_ = 9.689, *p* = 0.004) and strategy (trials correct: *F*_(35, 1)_ = 6.450, *p* = 0.016; blocks learned: *F*_(35, 1)_ = 10.678, *p* = 0.002; and stable trials: *F*_(35, 1)_ = 7.149, *p* = 0.011) on all three measures of VPM performance, as well as significant interactions between the two (trials correct: *F*_(35, 1)_ = 6.709, *p* = 0.014; blocks learned: *F*_(35, 1)_ = 8.133, *p* = 0.007; and stable trials: *F*_(35, 1)_ = 7.293, *p* = 0.011). *Post-hoc* tests revealed that older people performed worse specifically during place blocks (trials correct: *t*_35_ = 3.189, *p*_*B*_ = 0.003; blocks learned: *t*_35_ = 3.485, *p*_*B*_ = 0.002; and stable trials: *t*_35_ = 3.287, *p*_*B*_ = 0.002; Figure 3), accounting for the main effects of age and strategy, as well as for the interaction between them. This seems to suggest that older people may exhibit an allocentric strategy deficit.

**Figure 4:**
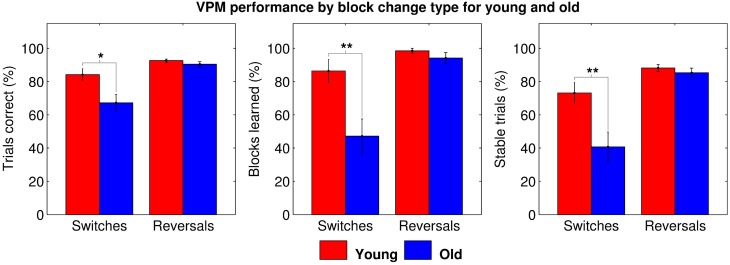
**VPM performance in terms of trials correct (left), blocks learned (middle), and stable trials (right) during blocks following switches and reversals for young (red) and old (blue) participants, excluding data from first blocks and blocks following unlearned blocks**. Significant group differences are indicated at the ^*^0.05, ^**^0.01, and ^***^0.001 levels following Bonferroni correction.

However, we then performed further ANOVAs with change type as the within-subjects factor, including data for blocks following switches and blocks following reversals in separate conditions. Data from blocks following unlearned blocks had to be excluded, as, even if that block was learned, it may not have necessitated a strategy switch or reversal. Data from the first block was also excluded as it of course preceded all changes. These gave similar results, again demonstrating a significant main effect of age (trials correct: *F*_(35, 1)_ = 8.083, *p* = 0.007; blocks learned: *F*_(35, 1)_ = 9.790, *p* = 0.004; and stable trials: *F*_(35, 1)_ = 8.441, *p* = 0.006), and also a significant main effect of change type (trials correct: *F*_(35, 1)_ = 23.112, *p* < 0.001; blocks learned: *F*_(35, 1)_ = 20.976, *p* < 0.001; and stable trials: *F*_(35, 1)_ = 26.915, *p* < 0.001) and a significant age by change type interaction (trials correct: *F*_(35, 1)_ = 5.074, *p* = 0.031; blocks learned: *F*_(35, 1)_ = 7.277, *p* = 0.011; and stable trials: *F*_(35, 1)_ = 6.638, *p* = 0.014) for all three dependent variables. *Post-hoc* tests revealed that older people performed worse specifically for blocks following strategy switches (trials correct: *t*_35_ = 2.667, *p*_*B*_ = 0.012; blocks learned: *t*_35_ = 3.043, *p*_*B*_ = 0.004; and stable trials: *t*_35_ = 2.878, *p*_*B*_ = 0.007; Figure 4), accounting for the effects of age and change type and the interaction between them. This seems to suggest that older people are impaired at strategy switching, rather than simply at employing allocentric strategies.

**Figure 5:**
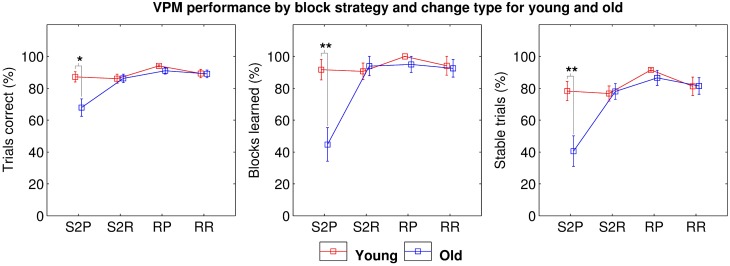
**VPM performance in terms of trials correct (left), blocks learned (middle), and stable trials (right) during blocks following switches to the place strategy (S2P), switches to the response strategy (S2R), reversals of place (RP), and reversals of response (RR) for young (red) and old (blue) participants, excluding data from first blocks and blocks following unlearned blocks**. Significant differences are indicated at the ^*^0.05, ^**^0.01, and ^***^0.001 levels following Bonferroni correction.

Taken together, these results suggest that either older people exhibit two separate general deficits in allocentric strategy use and strategy switching, which both contribute to impaired VPM performance, or they exhibit a more specific deficit in switching to an allocentric strategy. We assessed these two hypotheses by separating the data into four block types; those following a switch to the place strategy (S2P), those following a switch to response (S2R), those after a reversal of place (RP) and those after a reversal of response (RR). Further mixed ANOVAs with block type as the within-subjects factor revealed a significant main effect of age (trials correct: *F*_(35, 1)_ = 8.949, *p* = 0.005; blocks learned: *F*_(35, 3)_ = 9.486, *p* = 0.004; and stable trials: *F*_(35, 3)_ = 8.177, *p* = 0.007) and block type (trials correct: *F*_(35, 1)_ = 9.762, *p* < 0.001; blocks learned: *F*_(35, 3)_ = 9.927, *p* < 0.001; and stable trials: *F*_(35, 3)_ = 9.755, *p* < 0.001) as well as a significant interaction between the two (trials correct: *F*_(105, 3)_ = 5.702, *p* = 0.001; blocks learned: *F*_(105, 3)_ = 6.773, *p* < 0.001; and stable trials: *F*_(105, 3)_ = 5.570, *p* = 0.001). *Post-hoc* tests clarified that this was due to a more specific deficit in switching to place blocks (trials correct: *t*_33_ = 2.895, *p*_*B*_ = 0.013; blocks learned: *t*_33_ = 3.634, *p*_*B*_ = 0.002; and stable trials: *t*_33_ = 3.192, *p*_*B*_ = 0.006; Figure 5) among the older participants.

**Figure 6:**
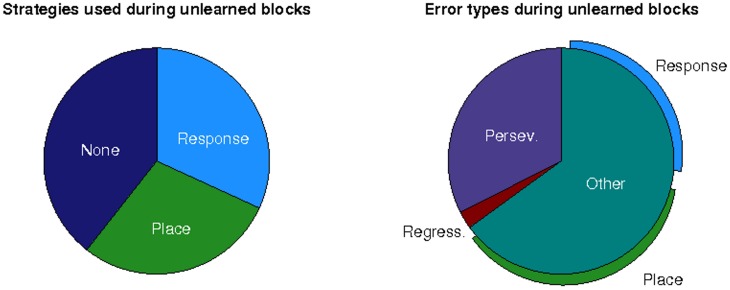
**Left:** Strategies used during unlearned blocks as a proportion of all unlearned blocks for old participants. **Right:** Error types made during unlearned blocks as a proportion of all unlearned blocks in which an incorrect strategy was used for old participants.

In an effort to understand why older participants failed to learn the place strategy following a switch, we explored the use of alternative strategies during unlearned blocks. For this analysis we simply associated each block with a particular strategy-direction combination if the participant responded in accordance with it significantly more than expected by chance (*p* < 0.001). As shown in Figure 6, while older participants often simply did not acquire any strategy, for the majority of the time they employed an incorrect strategy. Interestingly, the older participants used an incorrect place strategy just as often as an incorrect response strategy, providing evidence that they were able to use a place strategy just as well, despite the deficit in switching to this strategy (switch-to-place deficit). The older group also exhibited perseverative errors (continuing to use the strategy from the block preceding a change) in less than one third of blocks in which they used an incorrect strategy, suggesting that, in most cases, failure to learn the correct strategy was not simply due to failure to detect a change in reward contingency. There was very little incidence of regressive errors (changing back to the strategy used before the block preceding a change). Where other types of error were made, older participants again used both incorrect place and response strategies, suggesting that they were not simply reverting to a preferred responsestrategy.

We also assessed response times, but while the older participants generally took significantly longer to respond (*t*_35_ = 3.159, *p* = 0.003), this difference was consistent across all block types (switch to place: *t*_35_ = 2.721, *p*_*B*_ = 0.040; switch to response: *t*_35_ = 2.984, *p*_*B*_ = 0.021; RP: *t*_35_ = 3.315, *p*_*B*_ = 0.009; and RR: *t*_35_ = 2.577, *p*_*B*_ = 0.057). This suggests that the difference in response time was not related to differences in overall performance or the switch-to-place deficit reported above. A difference in response times could have affected performance due to the limited time (3 s) that participants had to respond, but very few participants from either group ever took longer than this to respond, and there was no significant difference between groups in the number of trials to which a response was not provided in time (*t*_35_ = 0.920, *p* = 0.364). Interestingly, response times did not increase significantly following a switch or reversal (*t*_35_ = 0.971, *p* = 0.335).

## Discussion

We demonstrated aging-impaired navigation using a VPM, with older participants responding correctly for significantly fewer trials, stably acquiring the correct strategy for significantly fewer blocks, and maintaining a stable strategy for significantly fewer trials. As expected, this impairment was related to decreased ability—as measured by numbers of trials correct, blocks learned and stable trials—to switch between navigational strategies, despite intact ability to perform reversals within strategies. However, further investigation revealed that this deficit was more specific than expected, applying only to switching in one direction; to the place strategy. There was a significant age difference in trials correct, blocks learned, and stable trials for place blocks following a switch, but none for post-switch response blocks, or for blocks of either strategy following a reversal. This specific effect also produced an apparent age difference in performance during place blocks but not response blocks, in contrast to our original hypothesis. We also explored the behavior of older participants during unlearned blocks, finding that while they often failed to acquire any strategy, they more often used an incorrect strategy, and that this was not usually attributable to perseverative or regressive error types.

Our initial results appeared to provide evidence of an age-related strategy switching deficit, as hypothesized and as found previously using set-shifting tasks (Moore et al., 2003; Gamboz et al., 2009; Young et al., 2010). Such a deficit can be interpreted in terms of the NA hypothesis of strategy switching (Aston-Jones and Cohen, 2005; Bouret and Sara, 2005), as LCNA dysfunction is observed in aging (Mouton et al., 1994; Li et al., 2001; Grudzien et al., 2007) and has been shown to impair strategy switching (Tait et al., 2007). During unlearned blocks, older participants did not usually exhibit perseverative errors, suggesting that any switching deficit would result from a failure of the LCNA system and PFC to engage a new strategy, rather than a failure of the ACC/OFC to detect a change in reward, or of the LCNA system to disengage the old strategy.

However, while we hypothesized a general strategy switching deficit, we found that the older participants did not exhibit an impairment in switching from the place to the response strategy. We also found an age difference in performance during place blocks, which may have indicated an allocentric processing deficit, as demonstrated previously in older animals (Frick et al., 1995; Kikusui et al., 1999; Begega et al., 2001). But similarly, this did not apply to place blocks following a reversal, and therefore did not suggest a general allocentric processing deficit. Instead, we found that these effects could both be attributed to a single specific impairment in switching to the place strategy. We believe this is the first study to identify this specific impairment, but we propose that previous findings interpreted as evidence of a general strategy switching deficit may actually relate to this more specific deficit. Furthermore, impaired switching to allocentric processing is likely to affect navigation in general, and may account for previous findings of age-related decline in allocentric navigation performance.

A strategy switching deficit could be attributed to LCNA or PFC dysfunction (Aston-Jones and Cohen, 2005; Bouret and Sara, 2005), whereas an allocentric processing deficit would most likely be attributed to hippocampal atrophy (Driscoll et al., 2003; Du et al., 2003, 2006). However, the cohort of older participants used in this study appear to have retained normal functionality of the areas of PFC and hippocampus responsible for co-ordinating switching and allocentric navigation, respectively. The specific switch-to-place deficit observed may instead stem from a functional difference somewhere between these two systems in the network involved in guiding navigation. We speculate that the interaction between hippocampus and PFC may be compromised in the older participants. In this case, when a response strategy is no longer rewarded, a switch would still be initiated by the LCNA system and the hippocampal place strategy would still be available to switch to, but the PFC would fail to select the place strategy as the best to use, perhaps due to reduced weighting of inputs from the hippocampus. If the weighting of caudal inputs is retained, then switches to response should be unaffected. Similarly, once a place strategy has been selected, although this will be more difficult, use of the strategy and performance of reversals should also remain unimpaired. There is some existing evidence of a change in the functional connectivity between the hippocampus and PFC in aging and dementia (Grady et al., 2003; Wang et al., 2006; Bai et al., 2009).

Reduced hippocampal-prefrontal connectivity could also account for other aspects of navigational aging, such as a preference among older people for egocentric strategies (Rodgers et al., 2012; Wiener et al., 2012). We recently demonstrated that this preference persists even when an allocentric strategy would be more beneficial to task performance (Wiener et al., submitted). This may result from older people's inability to switch to an allocentric strategy, due in turn to reduced hippocampal-prefrontal connectivity. Alternatively, reduced hippocampal-prefrontal connectivity could effect this strategy preference directly. In this case, the egocentric preference may contribute to the switch-to-place deficit. In the present study, older participants may have found it easier to switch to the response strategy due to their egocentric strategy preference, thus masking the effects of a more general switching deficit on their switch-to-response performance. Unfortunately, this behavioral data does not allow us to easily discern whether the effects were due solely to an age difference in functional hippocampus-PFC connectivity underlying the switch-to-place deficit, nor whether there was additional dysregulation of the LCNA system, corresponding to a more general deficit in strategy switching in combination with an egocentric strategy preference. Further investigation using neuroimaging is necessary to confirm the functional, morphological, or structural changes responsible for the behavioral age differences reported here.

In summary, we have demonstrated a specific age-related deficit in switching to an allocentric navigational strategy, while both switching to an egocentric strategy and reversals within strategies were unaffected. This deficit is unlikely to result from diminished allocentric processing functionality of the hippocampus, as older participants were still able to use a place strategy, and sometimes did so even when incorrect. It is also unlikely to stem from an impairment in reward monitoring in the ACC or OFC, as most errors made by older participants were not perseverative. Instead, we propose that the deficit corresponds to a failure to engage the place strategy due to decreased functional connectivity between the hippocampus and PFC. Dysregulation in the LCNA system may also contribute to this deficit, either in combination with a preference for egocentric strategies, or simply because the hippocampus-PFC connection could be affected by norardrenergic input from LC. Whatever the underlying cause, the specific switch-to-place deficit may explain more general impairments in navigational strategy switching and allocentric processing that have been observed previously, and is likely to contribute to the age-related decline of navigation ability in general.

### Conflict of interest statement

The authors declare that the research was conducted in the absence of any commercial or financial relationships that could be construed as a potential conflict of interest.
